# Modes and model building in *SHELXE*

**DOI:** 10.1107/S2059798323010082

**Published:** 2024-01-01

**Authors:** Isabel Usón, George M. Sheldrick

**Affiliations:** a ICREA, Institució Catalana de Recerca i Estudis Avançats, Passeig Lluís Companys, 23, Barcelona, E-08003, Spain; b Crystallographic Methods, Institute of Molecular Biology of Barcelona (IBMB-CSIC), Barcelona Science Park, Helix Building, Baldiri Reixach, 15, Barcelona, 08028, Spain; c Department of Structural Chemistry, Georg-August Universität Göttingen, Tammannstrasse 4, 37077 Göttingen, Germany; ESRF, France

**Keywords:** model building, phasing, density modification, MRSAD, *SHELXE*

## Abstract

Density modification is a standard step to provide a route for routine structure solution by any experimental phasing method, with single-wavelength or multi-wavelength anomalous diffraction being the most popular methods, as well as to extend fragments or incomplete models into a full solution. The effect of density modification on the starting maps from either source is illustrated in the case of *SHELXE*. The different modes in which the program can run are reviewed; these include less well known uses such as reading external phase values and weights or phase distributions encoded in Hendrickson–Lattman coefficients. Typically in *SHELXE*, initial phases are calculated from experimental data, from a partial model or map, or from a combination of both sources. The initial phase set is improved and extended by density modification and, if the resolution of the data and the type of structure permits, polyalanine tracing. As a feature to systematically eliminate model bias from phases derived from predicted models, the trace can be set to exclude the area occupied by the starting model. The trace now includes an extension into the gamma position or hydrophobic and aromatic side chains if a sequence is provided, which is performed in every tracing cycle. Once a correlation coefficient of over 30% between the structure factors calculated from such a trace and the native data indicates that the structure has been solved, the sequence is docked in all model-building cycles and side chains are fitted if the map supports it. The extensions to the tracing algorithm brought in to provide a complete model are discussed. The improvement in phasing performance is assessed using a set of tests.

## Abstract 2

All alternative *SHELXE* modes, using single or combined sources of starting phase information, are described. Side-chain tracing now completes model building in *SHELXE* to enhance density modification.

## 1. Introduction

Starting phases from molecular replacement (MR) or experimental phasing (see, for example, Read, 2001 ▸; Hendrickson *et al.*, 1985 ▸) are often not accurate enough to make the solution of a macromolecular structure evident and to allow the building a complete model for refinement. Still, once a starting solution is obtained it is possible to constrain the electron density to conform to previous structural knowledge. Upon back-transformation of the modified map, combination with the transformed phases rendered is used to improve the original phases. Such procedures are called density modification and were pioneered for macromolecules by Main (1967 ▸), while the first successful application of density modification was reported for small molecules by Hoppe and Gassmann in their phase-correction method (Hoppe & Gassmann, 1968 ▸).

Many sophisticated density-modification schemes have been proposed and have been incorporated into widely used programs such as *DM* (Cowtan & Main, 1998 ▸), *SOLOMON* (Abrahams & Leslie, 1996 ▸) and *RESOLVE* (Terwilliger, 2000 ▸). Effective concepts for macromolecular density modification include noncrystallographic symmetry (NCS) averaging (Main, 1967 ▸; Bricogne, 1976 ▸; Kleywegt & Read, 1997 ▸), solvent flattening (Wang, 1985 ▸), histogram matching (Zhang & Main, 1990 ▸), solvent flipping (Abrahams, 1997 ▸) and statistical approaches (Terwilliger, 2000 ▸, 2003 ▸; Cowtan, 2000 ▸). Partial structure interpretation is extremely powerful as it extends information over the whole resolution range and is widely used, for example in the *ARP*/*wARP* algorithms (Perrakis *et al.*, 2001 ▸).

*SHELXE* implements an alternative approach aiming to enforce stereochemical knowledge, the sphere-of-influence algorithm (Sheldrick, 2002 ▸), which is iterated with main-chain tracing (Sheldrick, 2010 ▸). In *SHELXE*, map interpretation has now been extended into the side chains to improve the phases and provide a more complete model.

## 2. Density modification in *SHELXE*

### 2.1. General principles

**Figure 1:**
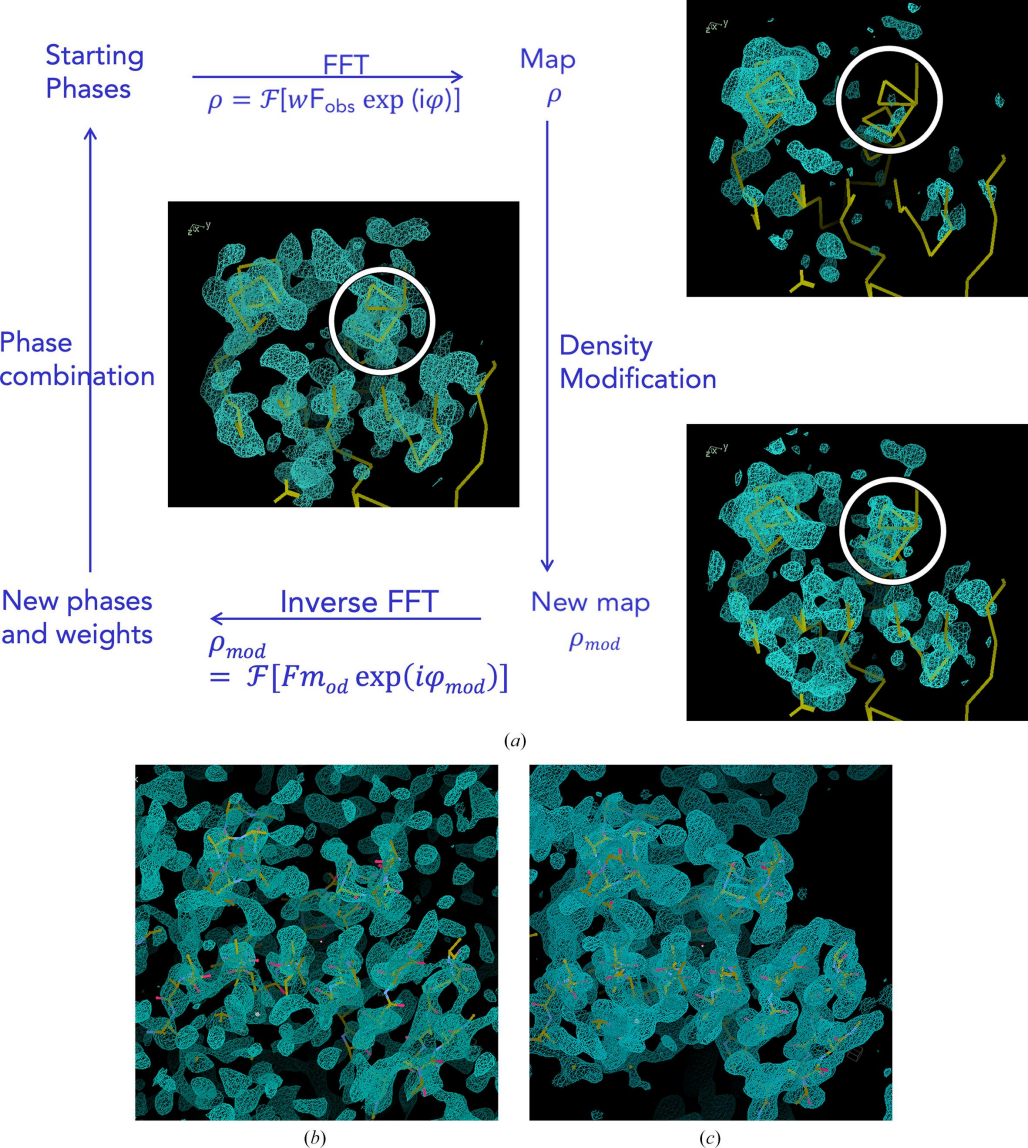
The density-modification process (Zhang *et al.*, 2001 ▸). (*a*) The process exemplified by the extension of a structure from the phases provided by a placed polyalanine helix in the structure of the C-terminal domain of Spr1875 at 1.6 Å resolution. For comparison, maps for the same protein in a different crystal, containing four zinc sites (PDB entry 6ysd), are shown. Data collected at a wavelength of 1.28224 Å to a resolution limit of 1.5 Å are used. (*b*) The map calculated with raw SAD phases shows noisy electron density everywhere. (*c*) The final density-modified map derived from that shown in (*b*).

Fig. 1 ▸ shows a scheme representing phase improvement by density modification, adapted from the relevant chapter in *International Tables For Crystallography* (Zhang *et al.*, 2001 ▸), to illustrate its practical effect in *SHELXE*. The idea is general, whether the starting phases have been determined through experimental phasing, nowadays most frequently single-wavelength anomalous diffraction (SAD) or multi-wavelength anomalous diffraction (MAD), or calculated from a partial model placed by molecular replacement or through a combination of both sources, as in MRSAD (Panjikar *et al.*, 2009 ▸). Once approximate phases are available for a structure, an electron-density map can be computed. This density can be modified based on assumptions of the general physical properties underlying structure: for instance, that in X-ray diffraction density should never be negative (Karle & Hauptman, 1964 ▸). Prior knowledge and statistical analysis of its properties are brought into the process. The modified map can be inverted back to calculate structure factors and the resulting phases are expected to have improved. Combining them with the original phases and the experimental amplitudes, a new, presumably better, map is calculated to initiate a fresh iteration. Fig. 1 ▸ illustrates the effect of density modification in the case of a protein originally phased using *ARCIMBOLDO* (Millán *et al.*, 2015 ▸), displaying helices decorating a central β-sheet and containing zinc sites (PDB entry 6ys7). Initial phases are calculated from a single polyalanine helix of 16 amino acids placed by *Phaser* (McCoy *et al.*, 2007 ▸), emphasized in the original map as it provides the only clearly defined feature. As the phases are calculated from this helix, the resulting map would in any case show such helical density due to model bias (Brünger, 1997 ▸; Luebben & Gruene, 2015 ▸; for an animated illustration see https://chango.ibmb.csic.es/resource/colibri.html), even if the true structure does not contain a helix in this position. The map produced after 20 cycles of density modification has developed new features in areas where no initial model was present: in particular, clear density is apparent for a second helix, whereas interpreting the central β-sheet would be more challenging. Eventually, the map after additional model building followed by fresh cycles of density modification becomes very clear, with correct density extending to the side chains. Fig. 1 ▸(*b*) shows the map calculated for the same protein in a different, zinc-containing crystal (PDB entry 6ysd) from the raw SAD phases, after resolving the twofold ambiguity and adding the heavy-atom contribution. In contrast to the initial, model bias-dominated, fragment-derived maps in Fig. 1 ▸(*a*), the signal is more evenly distributed and noise is present everywhere in the map. Fig. 1 ▸(*c*) displays the resulting map for this data set after density modification and main-chain tracing.

### 2.2. Initial phase information: the different modes in *SHELXE*

In practice, *SHELXE* supports several sources of phase information as input, be it from an external calculation with a different program or internally generated and combined. Experimental phasing can be exploited for single isomorphous replacement with anomalous scattering (SIRAS), single isomorphous replacement (SIR), radiation-damage-induced phasing (RIP), MAD or, as in the case summarized in Table 1 ▸, SAD, after data preparation with *SHELXC*. Multiple isomorphous replacement (MIR) or MIR with anomalous scattering (MIRAS) would require an external program, such as *SHARP* (Vonrhein *et al.*, 2007 ▸). Alternatively, phases can be calculated from a (partial) model provided in orthogonal (PDB) or crystal coordinates placed by MR (Thorn & Sheldrick, 2013 ▸). Map coefficients, phases and weights in the *SHELXE* format .phi (renamed the .phs format), structure factors in .fcf format, generated with *SHELXL* (Sheldrick, 2015 ▸), or phase distributions encoded as Hendrickson–Lattman coefficients (Hendrickson & Lattman, 1970 ▸) constitute suitable input. Experimental and map or model phases can be combined, either providing a substructure located, for example, with *SHELXD* (Usón & Sheldrick, 1999 ▸) or *ANoDe* (Thorn & Sheldrick, 2011 ▸) or through an internal cross-Fourier synthesis and peak search. If the substructure is given, *SHELXE* will refer it to the same origin as the model and invert it if necessary. It is also possible to perform density modification on the phases derived from the model or map and have *SHELXE* determine the substructure in the final cycle. This last procedure would not combine both sources of phase information but may be useful in the context of some *CCP*4 pipelines (Agirre *et al.*, 2023 ▸), for a subsequent iteration or to identify correctly placed partial models.

The various modes of running the program are illustrated for the case of an apoferritin structure (PDB entry 2g4h; Mueller-Dieckmann *et al.*, 2007 ▸). Results are summarized in Table 1 ▸. The single data set, to a resolution of 2 Å, contains anomalous signal from the cadmium cations present. A helical polyalanine fragment of 32 residues was placed with *Coot* (Emsley *et al.*, 2010 ▸) as a partial model fragment to provide phases from the derived coordinates and temperature factors, map and structure factors. From the results shown in Table 1 ▸, it can be seen that the substructure used in the SAD experiment, reflecting the sites and occupancies in the deposited PDB entry, can be improved for phasing purposes. Actually, better results are obtained with fewer atoms, and the modes locating the substructure from the partial model or map return the four major cadmium sites. *SHELXE* can also refine the anomalous substructure provided (-z). In addition, it is possible to read in phase distributions, as shown in the table for this example, which were generated with *SHARP* starting from the same substructure sites. Anisotropic refinement of the substructure, accounting for various types of scatterers and starting from a phase distribution, may be required for difficult cases. The values in Table 1 ▸ show how this improved start leads to a much better map upon 20 cycles of density modification. In addition, this route will be necessary to solve a structure from a MIRAS experiment.

A partial model provides an alternative start, with slight differences between the PDB model and the map, as the map is used as provided, whereas the PDB model is trimmed to optimize the correlation to the native data of the structure factors calculated from it. Also, the default values for sharpening vary slightly for the orthogonal and crystal coordinate formats, due to their typical use contexts. MRSAD combination of experimental with model or map phases yields the best results. Any of these starts leads to the equivalent solved structure when model building is performed, and Table 1 ▸ shows the resulting phase errors when three cycles of main-chain tracing of the map are used to improve the phases.

### 2.3. The sphere-of-influence algorithm

Classical density modification, as established by B.-C. Wang (1985 ▸), divides the map into protein and solvent regions. Protein regions present the largest density fluctuations and their features should be further enhanced, whereas the density in the more featureless solvent region should be low and uniform and is accordingly flattened. Unique to *SHELXE*, the sphere-of-influence algorithm (Sheldrick, 2002 ▸) avoids locating and smoothing the boundary between the protein and the solvent. In this algorithm, the variance *V* of the density is calculated for a spherical surface of radius 2.42 Å (a typical 1,3 distance in a macromolecule) around each voxel in the map. For voxels with a low variance, as would be expected within the solvent region, the density at the voxel is ‘flipped’ (ρ′ = −γρ, where γ is typically 1.1 but may be set by the user). The procedure is related to the γ-correction (Abrahams, 1997 ▸), except that it does not require an explicit solvent boundary. For voxels with a high variance, typical for protein regions, the density is reset to zero if negative and is otherwise left unchanged or subjected to a sharpening function (ρ_mod_ = [ρ^4^/(ν^2^σ^2^(ρ) + ρ^2^)]^1/2^, with ν being resolution dependent and larger the higher the resolution). This function is similar in its behaviour to that used in *ACORN* (Foadi *et al.*, 2000 ▸). For intermediate values of the variance, *SHELXE* applies a weighted mean of the corrected values for the protein and the solvent regions. Sharpening is particularly effective at high resolution or for experimental phases, and in default use is downweighted for fragment-derived phases.

### 2.4. Extension of partial structures

**Figure 2:**
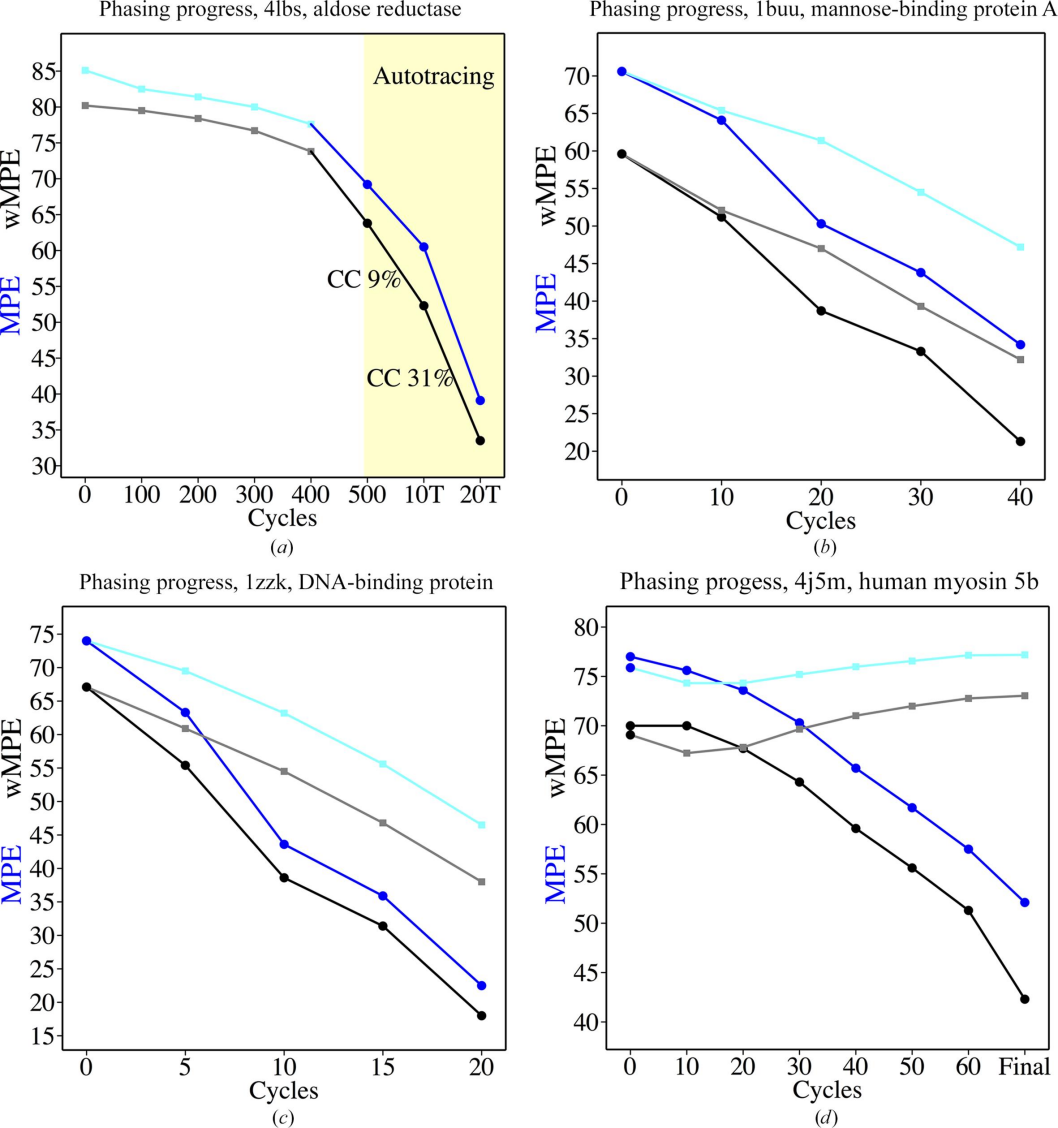
Mean phase error (blue lines) and weighted mean phase error (grey lines) starting from fragments and the role of model building. Dots show cycles with autotracing, while square points represent no autotracing. (*a*) Aldose reductase (PDB entry 4lbs; Fanfrlik *et al.*, 2013 ▸) at 0.76 Å resolution starting from the phases provided by the Br atom present in the ligand. The deposited structure contains 2567 protein atoms and 687 ligand and water non-H atoms. After 500 cycles of density modification a second run (yellow) iterates autotracing and density modification (ten cycles). (*b*) Mannose-binding protein A with 167 residues at 1.93 Å resolution (PDB entry 1buu; Ng *et al.*, 1998 ▸) from a holmium cation. The darker lines with dots (main-chain tracing) show a faster convergence and better final phases for the same total number of density-modification cycles than when no tracing is applied. (*c*) PDB entry 1zzk (Backe *et al.*, 2005 ▸) at 0.95 Å resolution, where a 82-residue structure can be obtained starting from four helical amino acids; autotracing is not essential but supports convergence. (*d*) Human myosin 5b (PDB entry 4j5m; Nascimento *et al.*, 2013 ▸) at 2.07 Å resolution, where main-chain interpretation is essential to extend from the two starting helices of 17 alanines to an interpretable map; without autotracing the phases deteriorate rather than improve.

Main-chain interpretation was incorporated into *SHELXE* to support phase improvement (Sheldrick, 2010 ▸) and was extended to include secondary-structure and tertiary-structure constraints for lower resolution purposes (Usón & Sheldrick, 2018 ▸). *SHELXE* typically traces one third to one half of the final structure in order to avoid compromising on accuracy, because deviations from the correct structure tend to quench the extension process. The factors underlying the chance of success in the extension of a partial structure with given diffraction data are well understood, if not predictable in a quantitative way. They are illustrated in Fig. 2 ▸ using four contrasting examples. The structure of aldose reductase (PDB entry 4lbs), shown in Fig. 2 ▸(*a*), in complex with the bromine-containing ligand {2-[(4-bromo-2,6-difluoro­benzyl)carbamoyl]-5-chlorophenoxy}acetic acid and NADP^+^ is, at 0.76 Å, one of the highest resolution structures deposited in the PDB (Fanfrlik *et al.*, 2013 ▸) for the comparatively large content of the asymmetric unit: 316 amino acids. Nevertheless, the Br atom, which can be placed from the native Patterson (Patterson, 1935 ▸), suffices to expand 85% of the structure. Fig. 2 ▸(*a*) illustrates how although the initial phases are characterized by an extremely high weighted mean phase error (wMPE), 

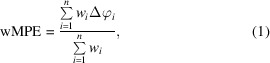

of above 80°, even density modification alone succeeds in very slowly improving the phase information, so that after 500 cycles the average phase errors have decreased by ∼5°. Main-chain autotracing accelerates convergence, and two rounds, interspersed with ten cycles of density modification, bring the errors down to 35°. Subsequent density modification brings the wMPE down to 15° (not shown in the figure). This constitutes a residual difference, given that the final deposited structure used as reference contains a model accounting for features established in the course of a high-resolution refinement that are outside the scope of the model used in phasing: H atoms, (anisotropic) displacement parameters, multiple conformations for disordered regions, bulk-solvent correction and scaling. Phase differences to the deposited structure are therefore never expected to be zero after density modification.

The resolution yielded by these aldose reductase crystals is extremely unusual, so in contrast PDB entry 1buu exemplifies a structure diffracting to a more typical resolution of 1.93 Å which can also be extended from a single atom to its 150 independent amino acids. Solution starts from a holmium(III) cation to provide starting phases characterized by an already remarkably low wMPE of below 60° (Fig. 2 ▸ *b*). It should be remarked that holmium(III), with 64 rather than 36 electrons as in Br^−^, represents a considerable contribution to the total scattering. In Fig. 2 ▸(*b*), a steeper convergence can be observed during the 40 cycles displayed in the *SHELXE* run, where main-chain tracing is used along with density modification versus the run where only density modification is used. This difference becomes negligible if both processes are allowed to run for more cycles until convergence, as in this and many other cases the final result will be limited by the quality of the data rather than by the starting phase information.

Modern synchrotrons and in-house diffractometers should be able to extract useful anomalous signal for experimental phasing, rendering the first two examples somewhat academic. Nevertheless, in the absence of heavy atoms the same pattern can be seen. Four residues (barely an α-helix turn) suffice, when correctly placed, to phase PDB entry 1zzk (0.95 Å resolution). As seen in Fig. 2 ▸(*c*), in this case tracing accelerates convergence, even though in its absence the same very low overall errors are eventually reached when more cycles are performed (not shown in the figure). Incomplete models at medium resolution may require autotracing for extension into a full solution; 10–15% of the main chain is typically enough at resolutions around 2 Å, as exemplified in Fig. 2 ▸(*d*) in the case of human myosin 5B (PDB entry 4j5m; Nascimento *et al.*, 2013 ▸). This protein is 396 residues long, the data extend to 2.1 Å resolution and it can be phased from two helices of 17 residues each, whereas without building the main-chain model these starting phases deteriorate and no solution is achieved. Cases such as those described constitute frequent targets in pipelines such as *AMPLE* (Bibby *et al.*, 2012 ▸; Rigden *et al.*, 2018 ▸; Simpkin *et al.*, 2019 ▸), *MrBUMP* (Keegan *et al.*, 2018 ▸) or *ARCIMBOLDO_LITE* (Sammito *et al.*, 2015 ▸).

The availability of high-resolution data has so far been critical for the extension of features outside the placed partial structures when these constitute a very limited fraction of the content of the asymmetric unit. Some improvement is achieved by extrapolating unmeasured data, whether missing low-resolution data or reflections beyond the resolution limit (Usón *et al.*, 2007 ▸), in what has been named the ‘free lunch’ algorithm. This option is used to generate electron-density maps and can produce spectacular results for high resolution and/or high solvent content.

### 2.5. Phase information from predicted models

The advent of accurately predicted models frequently allows routine molecular-replacement solution of crystallo­graphic structures using *AlphaFold* (Jumper *et al.*, 2021 ▸) or *RoseTTAFold* models (Baek *et al.*, 2021 ▸), for instance exploiting the optimized tools and pipelines available in *CCP*4 (Simpkin *et al.*, 2023 ▸). *SHELXE* now provides a feature to allow systematic elimination of model bias: -V will exclude the area occupied by the starting model from tracing. This feature is used for verification (Caballero *et al.*, 2018 ▸) within *ARCIMBOLDO_SHREDDER* (Sammito *et al.*, 2014 ▸: Millán *et al.*, 2018 ▸), which was originally designed to identify and refine the closest fragments present in a remote homolog structure. Within its dedicated mode for predicted models (Medina *et al.*, 2022 ▸), combining traces from different fragments ensures that the resulting solution is derived only from the inferences of each of these fragments while the original model has been systematically eliminated.

### 2.6. No structure solution despite partially correct phase information

Borderline cases occur where despite partially correct start phases it may still not be possible to solve the structure by interpreting the experimental map, extending a partial structure or eliminating the errors in a correctly placed model that presents large geometrical differences to the target. Locating the anomalous substructure or solving the molecular-replacement problem is not necessarily equivalent to solving the structure. When the starting phases are not accurate enough, and the more so the poorer the resolution, the correct starting information cannot successfully be extended and nonrandom starts remain unsolved. Also, a high percentage of helical structure is advantageous versus predominantly β-structures. In such cases, the brute-force method implemented in *SLIDER* of probing all favourable side-chain assignments onto a trace can extract a solution (Borges *et al.*, 2020 ▸). Our experience with this program underlies the choices to extend the model towards the side chains in the *SHELXE* implementation. Other approaches such as the sophisticated combination of building and refinement developed over the last three decades in *ARP*/*wARP* (Chojnowski *et al.*, 2020 ▸) should also be mentioned here. In the case of *SLIDER*, we observed that in the absence of powerful hardware to support the arduous calculations associated with probing all possible side-chain assignments, simplified modes considering only aromatic and hydrophobic residues or even reducing every side chain to a serine (Schwarzenbacher *et al.*, 2004 ▸) occasionally allowed a complete solution to be obtained from a poor start.

### 2.7. Gamma extension and map probing

**Figure 3:**
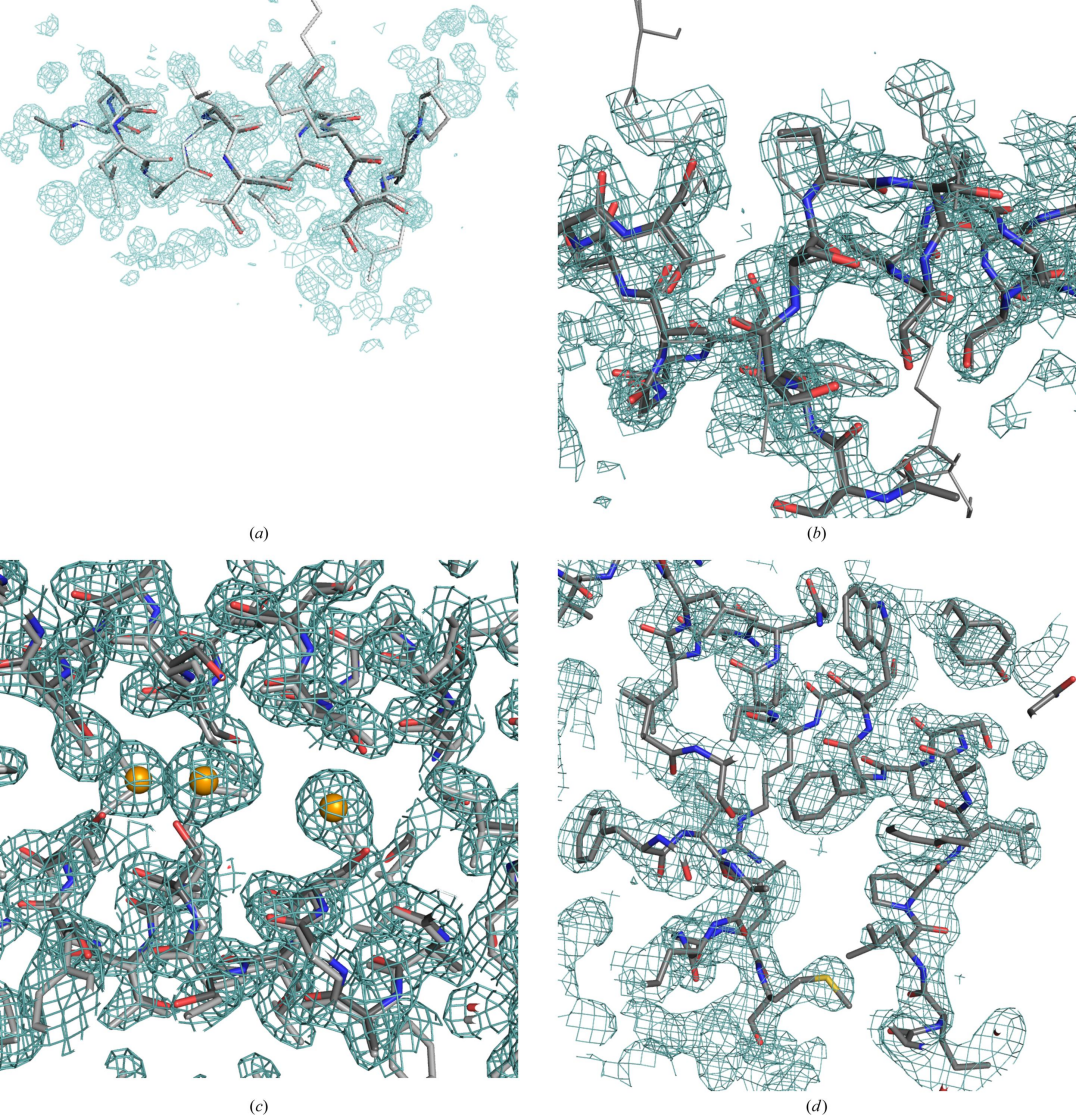
Tracing into the side-chain density. (*a*) Tracing of gamma positions illustrated in the first cycle for PDB entry 4ici, starting from experimental phases provided by two selenium sites. (*b*) Proline is identified from its density and position in the trace. (*c*) The use of substructure sites as sequence markers. (*d*) Traced model with side chains for CAS3.

Even in the absence of sequence information, it can be safely assumed that most residues will have a side chain with a C, O or S atom in the gamma position. As the density modification proceeds, main-chain electron density tends to be revealed earlier and more prominently, but even with large mean phase errors (see Fig. 1 ▸) clear electron density starts to show in parts of the structure. Gamma positions typically cluster in one of three staggered positions (Lovell *et al.*, 2000 ▸). Probing density in each of these at a compromise distance of 1.47 Å, between the shorter CB–OG distance in serine and the CB–CG distance in most amino acids, establishes whether the difference between the highest and lowest electron-density value is significant. Furthermore, it allows the detection of features in the map. In every autotracing cycle the trace is probed at the gamma position, provided that there is clear density for the beta position. Otherwise, the residue is annotated as a probable glycine. If there is clear discrimination between the highest and lowest density in the alternative position, the residue is modelled as pseudo-serine, with a slightly longer distance. Fig. 3 ▸(*a*) illustrates the inclusion of some gamma positions in the trace of a map for PDB entry 4ici, when it still has a large MPE of above 70°. If the maximum is not in *trans*, the ±30° conformation particular to proline will be probed with the appropriate geometry for its gamma carbon (Fig. 3 ▸ *b*). Finally, if the intermediate and highest density values are similar and clearly discriminated from the lowest valuee, the residue is annotated as a probable valine, threonine or isoleucine. Gamma sites are included in the calculation of trace-derived phases for the next cycle. The improvement that this provides is modest in the first cycle, less than 2° in the best cases, but in no case has it been found to deteriorate the phases, as Table 2 ▸ shows for a set of test cases.

### 2.8. Sequence docking

Sequence docking is performed by combining experimental evidence from the features in the current electron-density map and previous structural knowledge.

Sequence information is input through a FASTA format file named with the root name of the data file and the extension .seq. If the flag -O2 is set but no sequence file is provided, the program will issue a warning and will perform only gamma tracing to aid phasing, so that pipelines do not fail due to the lack of this file. To assign the corresponding sequence to each of the traced chains, these are considered from longest to shortest. Probabilities are then calculated for all possible sequence assignments obtained by sliding a copy of the sequence, with two trailing dummy residues on each end. Probabilities are calculated as the sum of the individual probabilities of each residue in the trace on a logarithmic scale, combining the score obtained probing the electron density and the probability derived from prior structural knowledge, following the scheme described in McCoy (2004 ▸).

If there is a substructure of anomalous scatterers comprising selenium from selenomethionine or sulfur from cysteine and/or methionine, its atoms are used as sequence markers (Fig. 3 ▸ *c*).

There is a vast amount of prior knowledge on sequence–structure relationships, but the reliability of secondary-structure prediction from the sequence is limited in the absence of comprehensive PDB data (Berman *et al.*, 2000 ▸; Lange *et al.*, 2020 ▸). It would be possible to involve the powerful methods implemented in *HHpred* (Söding *et al.*, 2005 ▸) as an external dependency, but rather than a comprehensive sequence analysis, *SHELXE* sequence assignment is guided by more robust principles, which stuck out clearly enough to be identified when only a very small subset of protein structures had been determined. Notably, the overall propensities of some amino acids to form secondary structure (Chou & Fasman, 1974 ▸), particularly the residues that typically terminate/initiate secondary-structure elements or marking loops (Richardson & Richardson, 1988 ▸; for example the proline displayed in Fig. 3 ▸ *b*), and the consistent association of hydrophobic residues upon sequence docking with the trace of a strand or a helix (Eisenberg *et al.*, 1984 ▸). Fundamentally, it is the available electron-density map that can be interrogated and the secondary structure of the trace internally described with characteristic vectors (Medina *et al.*, 2020 ▸).

### 2.9. Error correction

As sequence docking relies on correct main-chain tracing, a previous step to locate and remove connections with unusual Ramachandran values (Hollingsworth & Karplus, 2010 ▸) and lower density than that of flanking connections has been introduced to precede sequence docking. In general, to avoid errors the criterion used to accept an assignment is that it needs to be distinctly better than any alternative. Before incorporating side chains into the final trace (Fig. 3 ▸ *d*), a comparison of the correlation coefficient (CC, expressed as a percentage) calculated omitting the side chains for every stretch of chain is performed, analogous to the PDB optimization step introduced in *SHELX* macromolecular phasing (Sheldrick & Gould, 1995 ▸). If the CC characterizing the polyalanine trace is higher than when side chains are incorporated, they are eliminated from that part of the model.

## 3. Tracing tests

**Figure 4:**
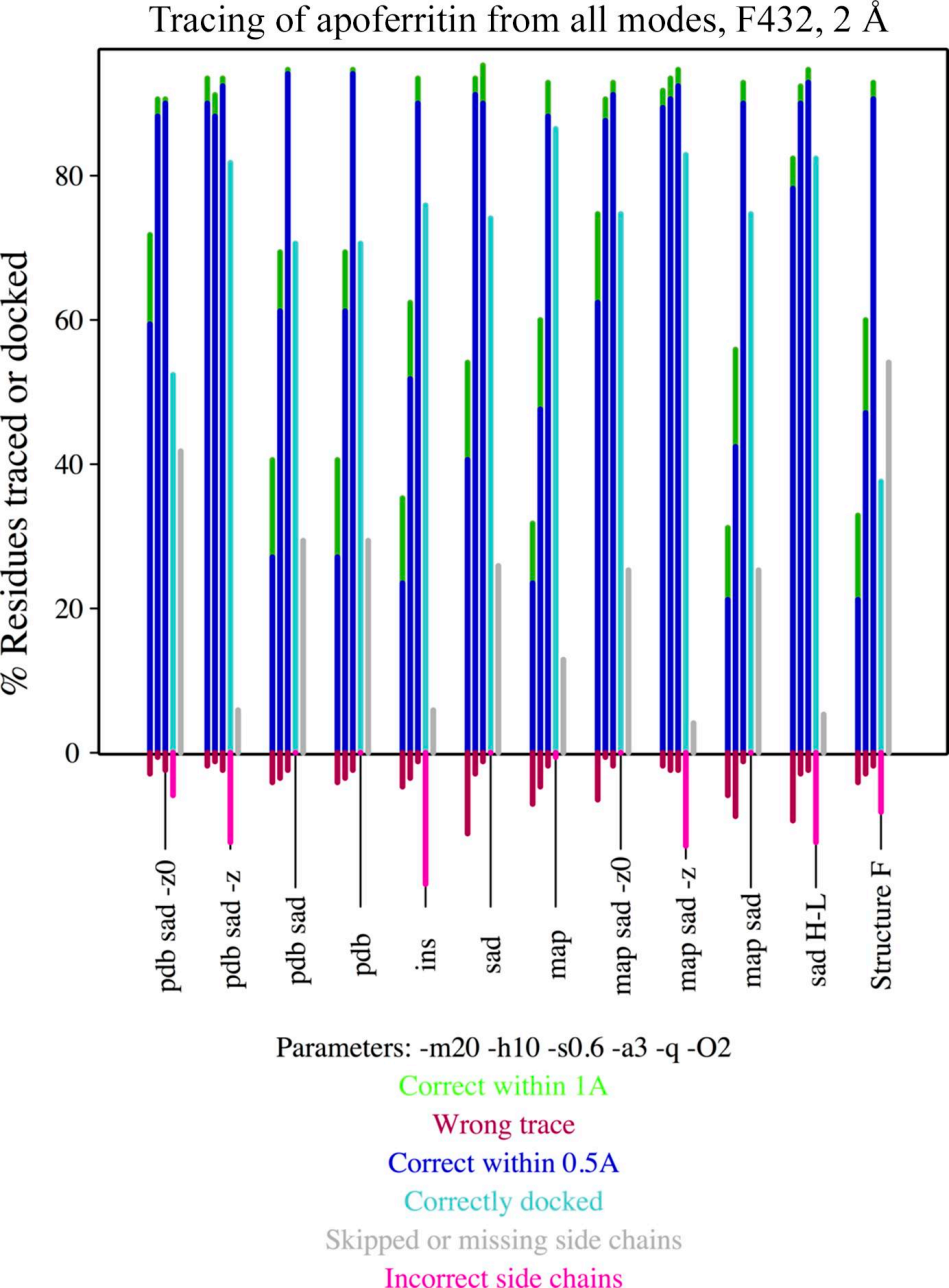
Percentage of the main chain correctly traced within 0.5 Å (blue), within 1 Å (green) and incorrectly traced (red) during the three autotracing cycles and performance of side-chain tracing in the final cycle as the percentage docked (cyan), skipped or absent from the main-chain trace (grey) and incorrectly traced (pink) for apoferritin (PDB entry 2g4h) starting from experimental, model or combined phases.

Fig. 4 ▸ displays the results of the phasing and model building of apoferritin (Mueller-Dieckmann *et al.*, 2007 ▸) described in Section 2.2. Starting phases are derived from anomalous difference data and cadmium sites or the phase-probability distributions calculated therefrom as Hendrickson–Lattman coefficients. Alternatively, starting phases of similar accuracy originate from a fragment of a long helix provided as a PDB file or in fractional crystal coordinates, as well as structure factors or a map calculated from the same model. Combinations of SAD and fragment or map phases are also included. The last model-building cycle involves side-chain tracing. In all of these cases, results after three groups of 20 cycles of density modification interspersed with map tracing are comparable. Nevertheless, in the cases where more complete starting information, combining model/map and SAD phases, is used convergence is faster and a more complete trace is already present in the first cycles.

Furthermore, 11 structures with resolutions ranging from 1.2 to 2.0 Å have been used to test and illustrate the new side-chain tracing features in *SHELXE* described above. Table 3 ▸ summarizes the characteristics, parameterization and phasing results obtained by incorporating side-chain tracing in *SHELXE* for SAD phasing and fragment cases where the atoms in the substructure or starting structure can be used as markers, as well as when this is not the case. For molecular-replacement solutions, initial phases should be limited (with the parameter -y) to a resolution dictated by the r.m.s.d. and extended in the course of density modification; the more limited the lower the identity between template and structure. For fragment phases, this default should be changed to use the full resolution available as small fragments should be accurate to be able to solve a structure. Hirustasin (Usón *et al.*, 1999 ▸) and bucandin (Kuhn *et al.*, 2000 ▸) have each been extended from ten sulfurs; SAD data for insulin and glucose isomerase were measured from nonmerohedrally twinned crystals (Sevvana *et al.*, 2019 ▸). The SusD protein (PDB entry 3l22) was originally solved using a MAD experiment (Vollmar *et al.*, 2020 ▸); for the purpose of this study it was phased from the peak wavelength used as SAD data at a resolution of 1.9 Å. VirusX CAS3 (Freitag-Pohl *et al.*, 2019 ▸) was the first previously unknown structure where we used the *SHELXE* development version to build a model with side chains. The flavoprotein with PDB code 4ici in space group *I*4_1_ was incorporated as a test where space-group inversion has to be performed at (0.5, 0.25, 0.5) rather than at the origin, an operation that is performed when phases from the fragment and from the pre-calculated anomalous substructure need to be referred to the same origin. Proteinase K (Wang *et al.*, 2006 ▸) and AmiA (M. Alcorlo, M. R. Abdullah, S. Hammerschmidt & J. Hermoso, unpublished work) are proteins phased from fragments of homologs, placed and refined with *ARCIMBOLDO_SHREDDER* (Millán *et al.*, 2018 ▸). The first is a test case starting from a map, as *ALIXE* (Millán *et al.*, 2020 ▸) combines solutions in reciprocal space and outputs a set of phases and figures of merit. The second was originally solved with *ARCIMBOLDO_SHREDDER* and the test starts from a fragment.

**Figure 5:**
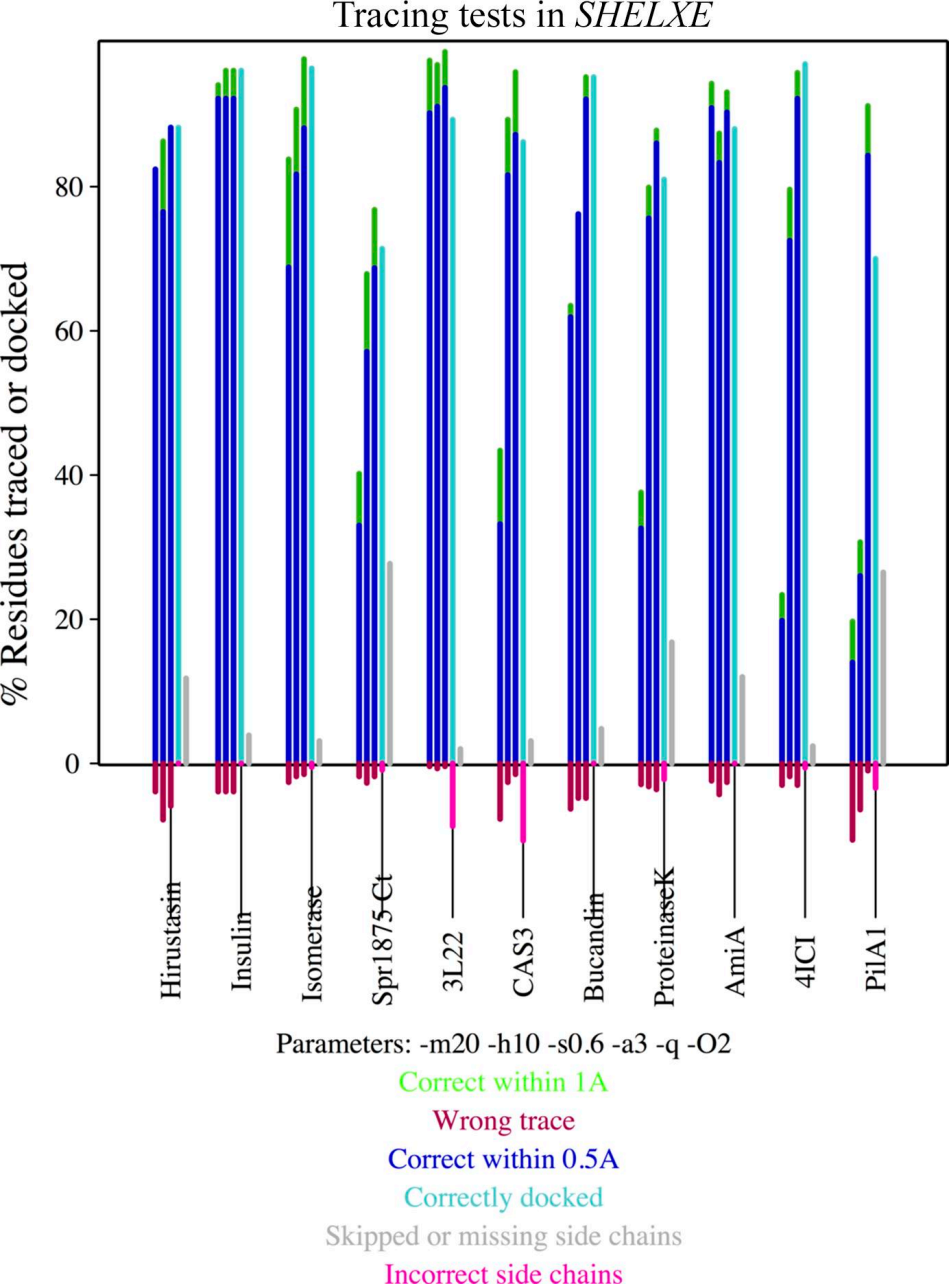
Percentage of the main chain correctly traced within 0.5 Å (blue), within 1 Å (green) and incorrectly traced (red) during the three autotracing cycles and performance of side-chain tracing in the final cycle as the percentage docked (cyan), skipped or absent from the main-chain trace (grey) and incorrectly traced (pink) for the structures summarized in Tables 2 ▸ and 3 ▸.

Finally, PilA1 (Crawshaw *et al.*, 2020 ▸) was originally solved with *ARCIMBOLDO_LITE* and contains three chains of 150 amino acids. For structures such as this one, with NCS, the FASTA format file read by *SHELXE* should explicitly contain a copy of the sequence for each of the chains present. The tracing results for all these cases are shown in Fig. 5 ▸.

## 4. Concluding remarks

This paper provides an overview of model building in *SHELXE* and of its effect as a constraint on density modification. It also describes all of the different modes in which *SHELXE* can be used and showcases how density modification can make a decisive contribution to phasing, which is sometimes hidden within pipelines.

The tracing algorithms have been expanded to enhance performance in borderline cases and to provide more complete models. Thus, the incorporation of side-chain atoms extends the phasing improvement brought about by model building. Furthermore, obtaining a more complete model, with side chains assigned and fitted to the density, is convenient. In the absence of a sequence or at lower resolution, tracing of polyserine has been added to *SHELXE* in all tracing cycles to increase model scattering. If a sequence is provided *SHELXE* can assign and fit side chains to the trace, which in the tests presented has been performed in the last tracing cycle, after extension of the gamma position in all previous cycles.

In view of the results presented, the recommended use, corresponding to the default triggered by the flag -O, when a sequence is provided is that gamma extension and density probing will be performed in every autotracing cycle, incorporating probable side chains for aromatic and hydrophobic residues with a partial occupancy of 0.6 into the trace used to generate phases for the next round of density modification. Still, models are output as polyalanine at this stage. Once the CC characterizing the trace reaches 30%, sequence docking will be performed in all remaining autotracing cycles, the best scored model with side chains will be saved as a PDB file and its derived phases will be combined in the calculation of the output map. *SHELXE* is often encountered within phasing pipelines, where a correlation coefficient of greater than 25% between the structure factors calculated from the polyalanine trace and the native data is adopted as an indication that the structure has been solved at a resolution of 2.5 Å or better. As seen in Table 3 ▸, CC values up to twice those typically rendered by main-chain tracing are obtained from the complete models. Therefore, the procedure implemented ensures that the CC value will be consulted in the polyalanine trace and that the most complete and correct model will be output incorporating side chains for a solved structure.

The performance of this procedure was assessed within the *Auto-Rickshaw* pipeline on a set of 40 structures that had not previously been used to develop the algorithms. The resolution in this pool of structures ranged between 2.0 and 2.4 Å, yielding improved results over the previous distributed version and nearly complete models.

At low resolution, model bias becomes a concern in the face of practically complete starting models. It is planned to extend the current feature (-V) to systematically exclude model bias.

## Figures and Tables

**Table 1:** *SHELXE* modes illustrated using a cadmium-containing apoferritin structure The data used are from Mueller-Dieckmann *et al.* (2007 ▸). In addition to the mode-specific parameters quoted in the table, all runs have a set 20 cycles of autotracing and 60% solvent content (-m20 -s0.6). A full list and description of parameters can be obtained by typing shelxe in a terminal. For main-chain autotracing, three cycles and the use of helical seeds were indicated as -a3 -q. Fragment optimization (-o) is used for .pda starts. As the data being phased contain cadmium, -h is shown whenever the anomalous substructure is used. All sites and occupancies for the cadmium cations were given as in the deposited PDB entry (2g4h). The tests include a map calculated from this same helical model in *SHELXE*, without further modification, and structure factors in .fcf format generated with *SHELXL* (Sheldrick, 2015 ▸). Hendrickson–Lattman coefficients were generated in *autoSHARP* (Vonrhein *et al.*, 2007 ▸) refining the sites and occupancies for the cadmium cations in the PDB entry.

				MPE; wMPE: all/centric (°)
Phasing information	Syntax	Parameters	Other files required	With main-chain tracing
Experimental (SAD/SIRAS/MAD)	a.phi a_fa	(-h)	a.hkl a_fa.{res, hkl}	66.8/67.3; 57.4/53.7
				39.6/42.6; 28.9/23.4
Model PDB	a.pda	–	a.hkl	72.6/73.8; 63.8/62.7
				35.7/35.8; 26.2/19.4
Model crystal coordinates .ins	a	–	a.{ins, hkl}	72.1/72.1; 63.6/61.6
				35.8/36.7; 26.0/19.3
Map coefficients	a.phi	–	a.{ins, hkl}	72.8/72.9; 64.6/63.2
				38.6/38.7; 27.9/20.0
Model + experimental	a.pda a_fa	(-h)	a.hkl	
Given substructure		-z0	a_fa.{res, hkl}	57.0/57.1; 45.6/39.3
				37.5/40.0; 27.8/22.4
Locate and use substructure		­z(n)	a_fa.{ins, hkl}	33.6/37.7; 24.5/20.2
				32.3/ 6.1; 23.5/19.0
Locate substructure		–	a_fa.{ins, hkl}	72.6/73.8; 63.8/62.7
				35.7/35.8; 26.2/19.4
Map + experimental	a.phi a_fa	(-h)	a.hkl	
Given substructure		-z0	a_fa.{res, hkl}	58.3/58.5; 47.1/42.3
				37.7/40.8; 27.9/22.7
Locate and use substructure		-z(n)	a_fa.{ins, hkl}	33.2/36.9; 24.2/19.9
				32.1/35.2; 23.4/18.6
Locate substructure		–	a_fa.{ins, hkl}	72.3/72.6; 64.2/63.2
				36.1/36.9; 26.3/19.3
Hendrickson–Lattman coefficients	a.hlc	–a.{ins, hkl}	a.{ins, hkl}	48.8/53.0; 39.1/38.2
				34.9/37.4; 27.0/22.7
Structure factors	a.fcf	–	a.hkl	72.5/72.4; 63.7/60.6
				38.3/37.6; 27.8/20.0

**Table 2:** Summary of the effect of gamma tracing on test structures CC is for the trace; wMPE is the weighted mean phase error for this trace. Parameters used in the *SHELXE* line: -m, number of density-modification cycles; -h, number of heavy-atom sites to use; -s, solvent fraction; -a, autotracing; -q, length of helix template, default 7; -K, retain input fragment; -y, derive starting phases from the model to the given resolution limit; -I, in cycle 1 only use the number of specified cycles and use extrapolated reflections if -e; -e, extrapolate unmeasured reflections to the given resolution limit.

ID	Start	Space group	*d* (Å)	CC, –/-O	Parameters	MPE (wMPE), –/-O (°)	Data from
Hirustasin	10S fragment	*P*4_3_2_1_2	1.2	46.8	-m25 -s0.4 -a1 -K	41.9 (35.1)	Usón *et al.* (1999 ▸)
				47.5		41.3 (34.7)	
Insulin	6S SAD	*I*2_1_3	1.54	37.4	-m10 -s0.4 -h -a1	53.8 (47.7)	Sevvana *et al.* (2019 ▸)
				33.3		51.4 (46.0)	
Isomerase	2Mn SAD	*I*222	1.56	32.9	-m10 -s0.49 -h -a1 -i	56.7 (49.7)	Sevvana *et al.* (2019 ▸)
				34.8		55.4 (48.5)	
Spr1875Ct	H14 fragment	*P*3	1.6	11.6	-m100 -s0.5 -a1 -B1	71.4 (63.9)	
				12.2		71.3 (63.8)	
PDB entry 3l22	12Se SAD	*P*4_3_22	1.9	39.5	-m15 -s0.45 -a1 -q -h	48.8 (43.2)	Vollmar *et al.* (2020 ▸)
				42.3		46.7 (40.0)	
CAS3	2h16 fragment	*P*4_3_32	1.9	19.1	-m15 -s0.5 -d1.9 -a1 -q	65.1 (57.7)	Freitag-Pohl *et al.* (2019 ▸)
				19.6		64.8 (57.5)	
Bucandin	10S fragment	*C*2	0.96	30.5	-m25 -s0.4 -a1 -K	56.5 (48.7)	Kuhn *et al.* (2000 ▸)
				30.7		55.9 (48.2)	
Proteinase K	Map	*P*4_3_2_1_2	1.27	12.8	-m15 -s0.5 -a1 -S3 -e1 -I15	70.3 (64.0)	Wang *et al.* (2006 ▸)
				13.4		69.9 (63.4)	
AmiA	Polyalanine fragment	*P*2_1_2_1_2_1_	1.2	35.0	-m50 -s0.5 -a1 -q -e1 -I50 -v0.1	45.0 (37.6)	
				36.8		43.5 (36.1)	
PDB entry 4ici	3Se SAD	*I*4_1_	1.4	2.6	4ici 4ici_fa -m30 -h -s0.4 -i -a1	82.0 (77.6)	
				2.9		82.0 (77.4)	
PilA1	PDB entry 2h30	*P*4_1_2_1_2	1.65	10.6	-m25 -s0.5 -a1 -q	76.4 (70.4)	Crawshaw *et al.* (2020 ▸)
				10.9		76.3 (70.4)	

**Table 3:** Summary of density modification and model building with *SHELXE* on test structures Parameters used in the *SHELXE* line: -m, number of density-modification cycles; -h, number of heavy-atom sites to use; -s, solvent fraction; -a, autotracing; -q, length of helix template, default 7; -K, retain input fragment; -y, derive starting phases from the model to the given resolution limit; -I, in cycle 1 only use the number of specified cycles and use extrapolated reflections if -e; -e, extrapolate unmeasured reflections to the given resolution limit. Data used are as in Table 2 ▸.

ID	Start	Space group	*d* (Å)	Time	CC	Parameters -a3 -O2 -t3	MPE/wMPE (°)
Hirustasin	10S fragment	*P*4_3_2_1_2	1.2	357	57.7	-m25 -s0.4 -K -f -y1.2	25.5/18.8
Insulin	6S SAD	*I*2_1_3	1.54	363	61.0	-h -s0.5	28.9/24.5
Isomerase	2Mn SAD	*I*222	1.56	2203	60.0	-h -s0.49 -i	32.0/26.0
Spr1875	H14 fragment	*P*3	1.6	817	32.7	-m30 -s0.5 -q -y1.6	38.4/31.5
PDB entry 3l22	12Se SAD	*P*4_3_22	1.9	3542	54.3	-s0.5 -s0.45 -q -h	25.5/19.2
CAS3	PDB entry 2h16 fragment	*P*4_3_32	1.9	1397	62.8	-m15 -s0.5 -d1.9 -q	25.4/19.1
Bucandin	10S fragment	*C*2	0.96	589	49.4	-m25 -s0.4 -K -y0.9	27.7/22.2
Proteinase K	Map	*P*4_3_2_1_2	1.27	2047	51.1	-m15 -s0.5 -v0 -S3 -e1 -I15 -y1.80	20.9/16.1
AmiA	Polyalanine fragment	*P*2_1_2_1_2_1_	1.2	11147	40.0	-m50 -s0.5 -q -I50 -e1 -v0.1	28.0/21.6
PDB entry 4ici	3Se SAD	*I*4_1_	1.4	1274	50.3	-m30 -h -s0.4 -i	27.8/22.1
PilA1	PDB entry 2h30	*P*4_1_2_1_2	1.65	4580	46.3	-m25 -s0.5 -q -y1.65	23.5/17.5
